# Deglycosylation of *Tropheryma whipplei* biofilm and discrepancies between diagnostic results during Whipple’s disease progression

**DOI:** 10.1038/srep23883

**Published:** 2016-03-30

**Authors:** Gilles Audoly, Florence Fenollar, Jean-Christophe Lagier, Hubert Lepidi, Didier Raoult

**Affiliations:** 1 Aix Marseille Université, URMITE, UM63, CNRS 7278, IRD 198, Inserm 1095, 13005 Marseille, France

## Abstract

Whipple’s disease is a systemic infectious disease associated with the bacterium *Tropheryma whipplei*. Numerous reports have presented puzzling discrepancies between diagnosis methods. We addressed this confusion using fluorescent *in situ* hybridization and immunofluorescence assays to evaluate 34 duodenal biopsies and 1 lymph node biopsy from Whipple’s patients. We showed the presence of bacteria in both CK20^+^ epithelial cells and CD68^+^ macrophages. Bacteria are found embedded in a biofilm hindering the detection of *T. whipplei*. Only after treatment of biopsies by glycosidases, co-localization of *T. whipplei* RNA/DNA with bacterial proteins was restored. Moreover, using 13 bronchoalveolar lavages and 7 duodenal biopsies, we found that hydrolysis of the biofilm weakened the bacteria, facilitated bacterial DNA extraction and improved the sensitivity of qPCR detection by up to 1000x opening new perspectives for diagnostic and scientific approaches.

Whipple’s disease (WD) is a systemic disease that predominantly affects the intestine and is associated with the presence of *Tropheryma whipplei* in macrophages^1^,^2^. The diagnosis of this disease is often carried out by a duodenoscopy with histological examination, including periodic acid-Schiff (PAS) staining in conjunction with PCR and immunochemistry. Histological studies of mucosal biopsy sections using standard PAS staining have detected massive infiltration of the lamina propria by foamy macrophages, a finding that has long been considered the pathognomonic hallmark of WD^3^.

Since the first description of a genetic procedure for the diagnosis of WD^4^, the culturing of *T. whipplei* and the sequencing of its genome^5^,^6^,^7^ have allowed for the development of specific molecular biology techniques, including PCR-based diagnosis. Furthermore, axenic culture of *T. whipplei*^8^ enabled isolation of multiple *T. whipplei* strains from many clinical samples^9^. This technique also allowed for the development of specific antibodies to uncover the presence of the bacteria in paraffin-embedded tissue sections or circulating macrophages^10^,^11^. Immunohistochemistry using mouse or rabbit polyclonal antibodies is a useful complement for PAS staining, as it may avoid false positive results found by PAS^12^. However, these diagnostic methods have demonstrated their limitations in opposing situations^13^,^14^. First, there are cases where healthy *T. whipplei* carriers have a positive PCR-based diagnosis from saliva, feces, and duodenal biopsy samples^15^,^16^. Conversely, when a WD patient begins antibiotherapy, PCR detection becomes negative soon after treatment. This result is the opposite of the slow clearance of bacterial DNA observed during treatment of infective endocarditis^17^. In contrast, discontinuing treatment for WD is associated quickly with relapse and a positive PCR-based diagnosis^18^. Moreover, in duodenal biopsies PCR detection is negative, while PAS staining and *T. whipplei*-specific immunohistochemistry are positive for months or years after the start of treatment^19^,^20^.

Scanning electron microscopy studies revealed a biofilm corresponding to an assemblage of surface-associated microbial cells that is enclosed in an extracellular polymeric substance to form their immediate environment. This matrix is thought to be responsible for the different staining characteristics of the intracellular versus extracellular forms of *T. whipplei*, as well as the surprising bacterial resistance against chemical treatment^21^. The biofilm might also play a role in the survival of virulent strains in the intra/extracellular compartment of the host. It is well established that bacteria within a biofilm are known to adopt a distinct physiological state, as well as employ specific attachment and detachment mechanisms, that could have profound implications for the clinical manifestations such as inflammation, antibiotics resistance, persistence or the spread of the infectious agents^22^.

The precise nature of the biofilm surrounding the *T. whipplei* is currently unknown, but the corresponding gene cluster involved in extracellular polysaccharide synthesis^5^,^6^, as well as *Wisp* genes coding for a 110-kD surface hyper-glycosylated protein, was associated with the formation of this biofilm^23^. The composition of the biofilm is likely involved in fooling the immune system because the lower immunological response in WD patients is mostly restricted to proteins associated with glycans^24^, while asymptomatic carriers and patients with chronic disease have a strong antibody response against bacterial antigens^25^.

Bacterial biofilms found in clinical samples contain complex polysaccharides, which retain components known to inhibit PCR-based diagnostics and provide mechanical/chemical resistance^26^,^27^,^28^. These factors led us to test the hypothesis that the biofilm surrounding *T. whipplei* hindered detection of this bacterium by molecular probes and antibodies. We investigated the role of bacteria-embedding glycans in macrophage vacuoles using different diagnostic methods. We tested the effects of glycosidase treatment on an *in vitro*-cultivated strain of *T. whipplei* and clinical samples from WD patients using fluorescent *in situ* hybridization (FISH), confocal analysis and PCR. The discrepancies between the different diagnostic methods were eliminated by simple treatment with glycosidases, which also resulted in improved assay sensitivity. Our results provided insight into the phenomenon leading to the discrepancy between these detection methods and improved diagnostic interpretation. Additionally, this work mainly focused on Whipple bacillus detection in duodenal biopsies, expanding our understanding of the pathogenesis of WD and the early stages of WD.

## Detection and localization of ***T. whipplei*** in paraffin embedded biopsies from patients with Whipple’s Disease

**Figure 1:**
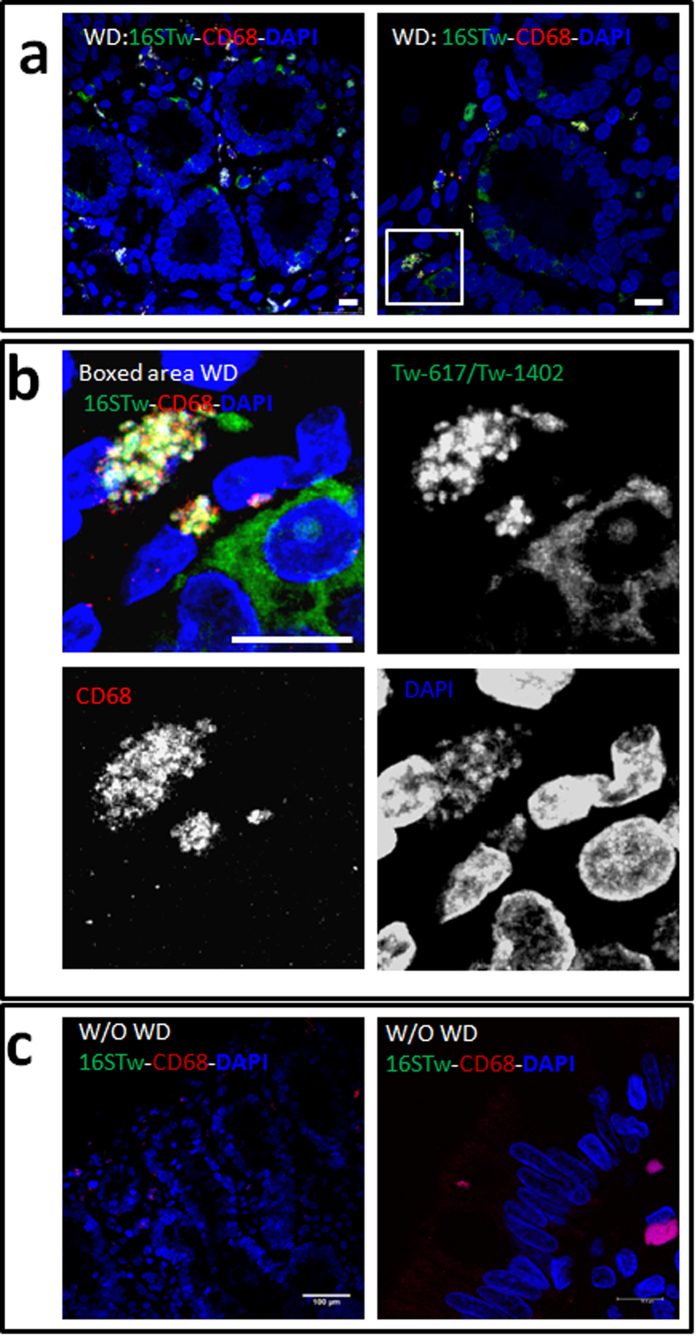
Detection and localization of *T. whipplei* in duodenal biopsy sections. (**a**,**b**) The sections of patients presenting Whipple disease (WD) or (**c**) an uninfected patient control without WD (W/O WD) were imaged using a confocal microscope, with the green channel (Alexa488) showing hybridizations of 16S rRNA probes Tw-617 and Tw-1402 and the red channel (Alexa550) showing immunostaining of CD68; The nuclei are counterstained with DAPI in blue. The white-boxed area of upper right image is magnified in the four images of panel (**b**) showing the merge image and the individual emission channels for each fluorophore. *T. whipplei* are detected in CD68^+^ compartment of macrophages. Some CD68^−^ cells, evocating epithelial cells, were also infected and present a diffuse cytoplasmic FISH signals showing free individual bacteria. FISH probes are concentrated in CD68^+^ vacuoles appear in yellow/white color surrounding nucleus, note that most of the bacterial DNA present in CD68^+^ vacuoles are stained by DAPI suggesting a high concentration of bacteria in phagosomes. No bacteria were detected in uninfected patient control without WD (W/O WD). Scale bars = 10 μm or indicated in the image.

We first conducted a series of FISH assays on 38 duodenal biopsies (DB), including 34 from WD patients undergoing therapy and 3 from unrelated patients. For FISH analysis, we used two different probes, Tw-617 and Tw-1402, which are complementary to regions within the *T. whipplei* 16S rRNA and match species-specific sequences. The confocal microscopy analysis of *T. whipplei-*specific probes bound to DB from the 34 WD patients, revealing that the bacteria were concentrated in relatively few cells and presented as positively DAPI-stained structures around the DAPI-stained nuclei (Fig. 1a,b, Supplementary Figures 1, 2). DB specimens with classical villous organization from 3 control patients without WD did not bind to *T. whipplei-*specific probes (Fig. 1c). We also used the previously described *T. whipplei* probes Tw-652 (Supplementary Figure 1), which complement a *T. whipplei*-specific region of 16S rRNA, and the universal Eub 338 probe, which targets most of the eubacteria (Supplementary Figure 2). These probes detected similar cell populations as those previously detected with Tw-617 and Tw-1402, confirming the presence of *T. whipplei* in our samples. From 18 biopsies, confocal microscopy established that the *T. whipplei* 16S rRNA probes co-localized with the *T. whipplei wisp* gene DNA probes (Supplementary Figure 1). Most of the signals of the DNA *wisp* genes matched with the 16S rRNA probes, suggesting that this portion of the bacteria are transcriptionally active and presumably targets viable bacteria, while a minor fraction of the bacteria detected only with *wisp* gene probes are dead or with an undetectable transcriptional activity (Supplementary Figure 1).

Furthermore, our confocal analyses revealed that FISH signals surrounded nuclei (Supplementary Figure 3) and co-localized with IF staining of CD68^+^ vacuoles (Fig. 1a,b). In merge images, the signals indicated morphology typical of infected macrophages with the accumulation of bacteria inside the CD68^+^ compartments (Fig. 1, Supplementary Figures 1, 2, 3).

**Figure 2:**
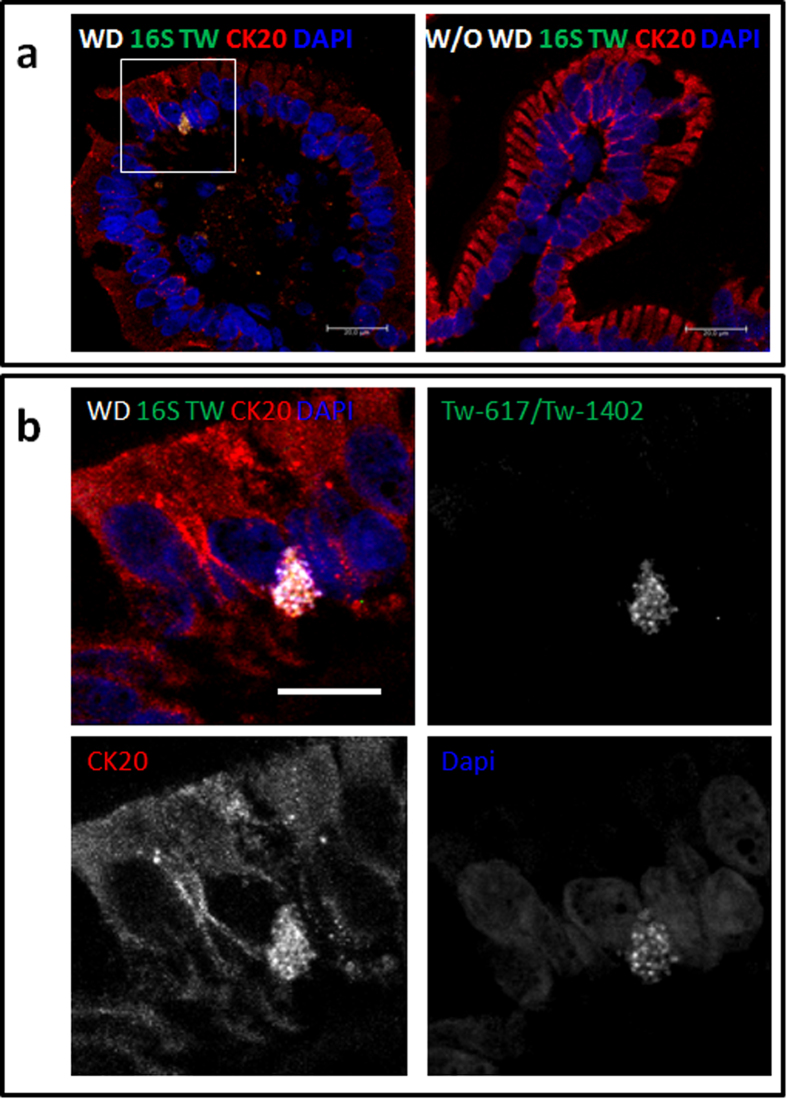
Confocal microscopy visualization of intracellular *T. whipplei* in epithelial cells of duodenal biopsy. Fluorescent *in situ* hybridizations of 16S rRNA probes Tw-617 and Tw-1402 and immunofluorescence of CK20 were done on duodenal sections of patients presenting Whipple disease (WD) or an uninfected patient control without WD (W/O WD). The images correspond to merged signals for RNA probes in green, associated with immunostaining for CK20 in red. Nuclei are counterstained with DAPI in blue. *T. whipplei* are detected in CK20^+^ epithelial cell at the tip of the villus and appeared concentrated in a punctuate CK20 rich region. The inset region of upper left image is magnified and presented in the bottom, showing individual emission channels for each fluorophore. FISH probes are concentrated in CK20^+^ regions appear in white color due to overlap areas labeled with DAPI. Scale bars = 10 μm.

Moreover, bacterial FISH probes revealed bacteria in the cytoplasm of some CD68 negative cells, located in the villi with a pyramidal morphology (Fig. 1, Supplementary Figure 4). Using the differentiation marker of the intestinal epithelium, cytokeratin 20 (CK20) and 16S rRNA probes we evidenced a productively infection in rare epithelial cells. The bacteria are concentrated in CK20 rich regions (Fig. 2, Supplementary Figure 4) or diffused in the cytoplasm of epithelial cells (Fig. 1b, Supplementary Figure 4). Few evidences of extracellular bacteria were found in our samples from WD patients undergoing therapy. No signal was detectable using the non-specific nonEub probe on samples from Whipple patients (Supplementary Figure 2). Taken together, these results demonstrated that both CD68^+^ compartment of macrophages and cytoplasm of CK20+ epithelial cells support the replicative form of the pathogen.

**Figure 3:**
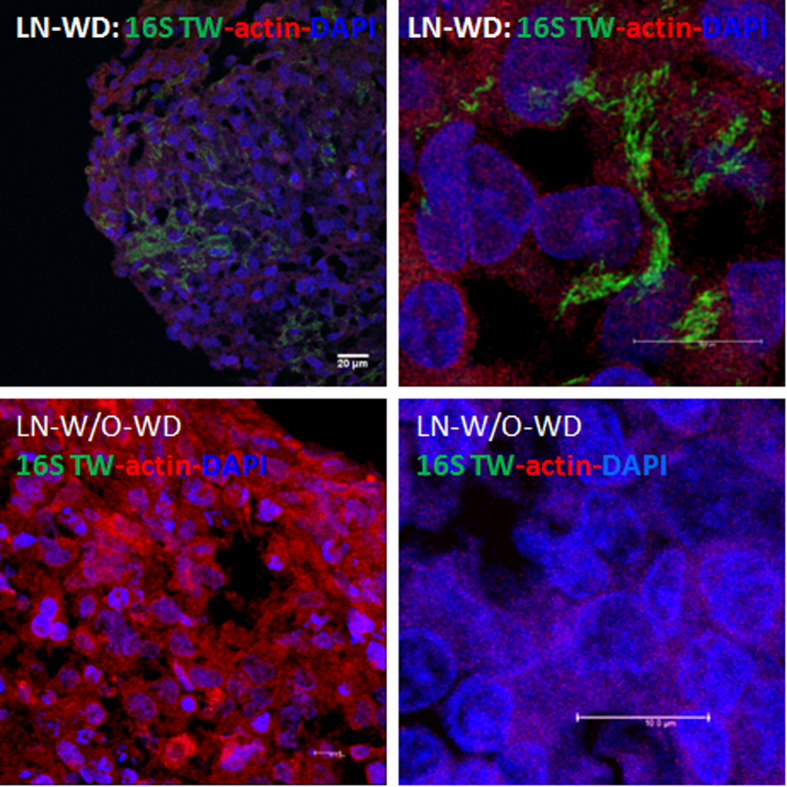
Confocal microscopy detection of *T. whipplei* in lymph node of patient presenting Whipple disease. Fluorescent *in situ* hybridizations of 16S rRNA probes Tw-617 and Tw-1402 in green channel, the F-actin staining in red were done on sections of patients presenting Whipple disease (WD) or an uninfected patient control without WD (W/O WD). The images correspond to merged signals for RNA probes in green, associated with phallacidin staining in red. Nuclei are counterstained with DAPI in blue. Scale bars are indicated on each image.

Moreover, a lymph node from a patient (A19) with Whipple’s disease (Supplementary table 8), prior an antibiotherapy, presenting a positive PCR to *whi2* gene in blood specimen was tested using FISH as previously done for duodenal biopsies. The sections of the lymph node bound 16S rRNA probes Tw-617 and Tw-1402, revealed long rope-like structures. At higher magnification, the bacteria are localized in areas sharing staining with the cytoplasmic F-actin surrounding cells (Fig. 3). The negative-control probe nonEub did not hybridize the lymph node sections. A lymph node from patient without Whipple’s disease did not bind 16S rRNA probes Tw-617 and Tw-1402. This approach allowed us to obtain a qualitative image of bacterial *T. whipplei* distribution in the lymph node.

### Discrepancies between conventional immunofluorescence and fluorescent *in situ* hybridization for the detection of *T. whipplei*

**Figure 4:**
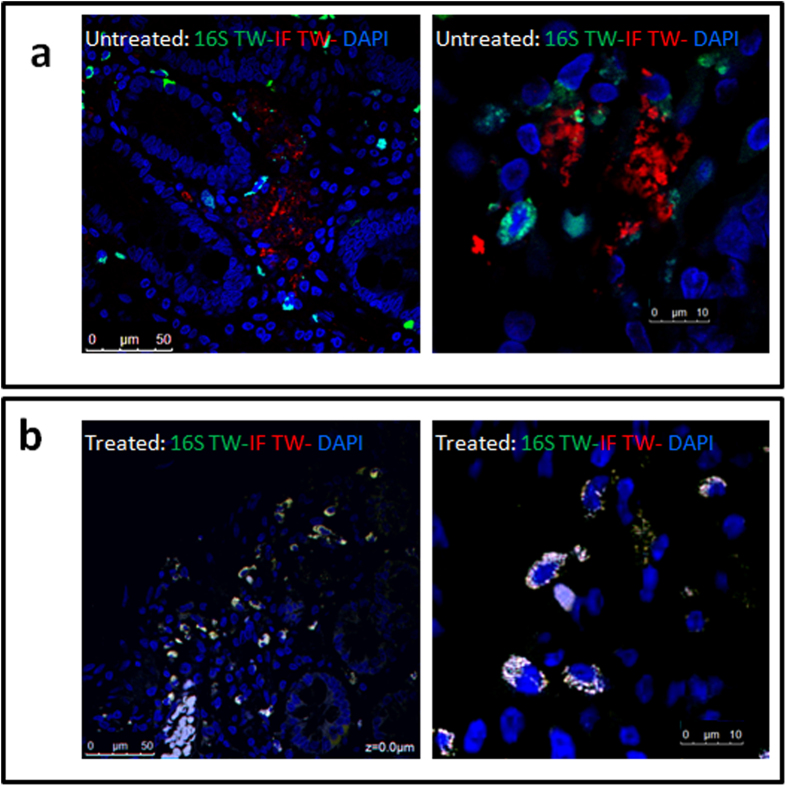
Deglycosylation restores an identical spatial distribution of *T. whipplei* FISH signals in positive area detected with specific IF of duodenal sections of patients presenting Whipple disease (WD). Confocal microscopy images illustrate the FISH 16S rRNA probes in green and the red IF staining by T. whipplei-specific antibodies in untreated samples with glycosidases (**a**) and samples treated with glycosidases (**b**). The co-localizations of FISH and IF signals were poorly observed in the untreated samples. In contrast, images from samples treated with glycosidases revealed the FISH in green overlapped with red IF staining by T. whipplei-specific antibodies leading to white signals indicating co-localization with DAPI staining. The regions of duodenal biopsy only stained by specific antibodies correspond to bacteria with degraded RNA or secreted *T. whipplei* antigens. These images are representative of observations for each condition. Bar scale are indicated in each image.

We next addressed the spatial association between bacterial rRNA and *T. whipplei* antigens by confocal microscopy. A FISH assay performed on 18 duodenal samples from patients presenting WD was followed by IF using a specific serum directed against *T. whipplei*. The IF revealed large positive regions in villi, lamina propria and deeper mucosal layers in the 18 biopsies, in contrast to the 16S rRNA probes, which mainly bound to discrete regions and was limited to only a few positive cells (Supplementary Figure 5). Higher magnification of the merge images of slides from Whipple’s patients demonstrated that many cells detected using *T. whipplei*-specific FISH probes were distinct from those detected with the *T. whipplei* antibodies (Fig. 4a). The majority of *T. whipplei*-specific IF staining failed to surround a nucleus stained with DAPI and FISH probes and may correspond to the ghost of dead, infected cells or extracellular *T. whipplei* antigens (Fig. 4a). Comparable discrepancies have been previously observed for FISH assays and PAS-reactive materials^29^. We hypothesized that these discrepancies were linked to the presence of a protective *T. whipplei* biofilm in infected cells that masked bacterial detection.

### Surface oligosaccharides of *T. whipplei* biofilm impairs detection by FISH/IF assays

We first evaluated the effect of different pretreatment protocols with detergents and/or protease to improve the bacteria detection in *T. whipplei-*infected MRC5 cells by unmasking target antigens and 16S rRNA. Neither pretreatment with SDS, triton x-100, or NP40, either alone or in combination with protease, nor elevated temperature, led to increased co-localization of FISH and IF signals relative to our previous results, only few intracellular bacteria are both revealed by FISH and IF. In contrast, pretreatment consisting of the hydrolysis of N- and O-link glycans allowed co-detection of most of bacteria in infected cells by 16S rRNA probes and antibodies (Supplementary Figure 6). Interestingly, by comparing the relative fluorophore signal intensity with background autofluorescence and exposure time, we found that the FISH signals following glycosidase treatment detected bacteria with a stronger staining than in untreated cells. The reactivity of antibodies to unglycosylated bacterial antigens was slightly lower in treated cells but remained sufficient to detect individual bacteria.

Based on this result, we further investigated the effect of N- and O-glycosidase activity on 18 tissue slides from patients with Whipple’s disease and 2 slides from patients without Whipple’s disease. Confocal microscopy analysis of all of the 18 WD biopsies was positive for FISH signal. The removal of carbohydrates improved detection by DNA/RNA-specific probes of infected cells (Fig. 4b). Most of infected cells showed that the DNA/RNA-specific probes co-localized with *T. whipplei* specific IF, corresponding to bacteria associated with vacuoles (Fig. 4b, Supplementary Figure 5). Some IF-positive regions within mucosa did not bound FISH probes, enlightening that the mucosa contains glycosylated, extracellular *T. whipplei* antigens (Supplementary Figure 5).

**Figure 5:**
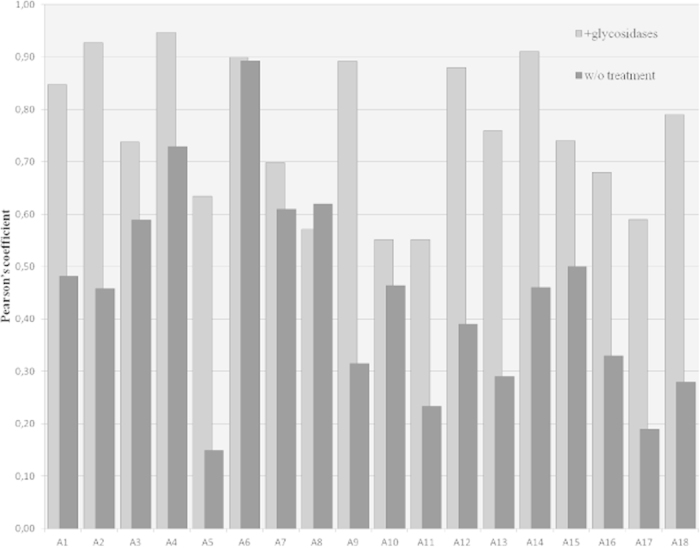
Quantification of co-localization of FISH and IF signals in 18 duodenal biopsies of WD patients after treatment with or without glycosidases. All samples were analyzed by fluorescence confocal microscopy, and each image was processed to determine the Pearson’s coefficient, which ranges from 0 (no co-localization) to 1 (perfect co-localization). To exclude random co-localization of pixels, the image thresholds for each fluorophore were automatically generated using the Coste’s system. Columns present data from confocal analysis of each patient (A1 to A18); for each patient, columns indicate the Pearson’s coefficient for the treated (light grey) and untreated (dark grey) samples.

We used software to quantify the co-localization of signals from confocal images with parameters which significantly reduce the pseudo-colocalization and ensure the reliability of calculations. As shown in Fig. 5, the Pearson’s coefficient from samples treated with glycosidases ranged from 0.55 to 0.95, corresponding to moderate to very strong correlation. Furthermore, the mean of the overall coefficients indicated strong co-localization (mean = 0.76 +/− 0.134). In contrast, the corresponding coefficient values from untreated samples were lower, ranging from moderate to weak co-localization (mean = 0.44 +/− 0.189) with 13/18 slides below 0.5. Only one sample showed a coefficient similar to that of a treated sample (0.89 vs 0.90). Overall, glycosidase treatment markedly improved co-localization of signal in macrophages, demonstrating that the glycan biofilm embedded the replicative form of *T. whipplei* and hindered bacterial detection.

### N- and O-glycans protect *T. whipplei* bacteria from lysis

We hypothesized that surface glyco-conjugates have a protective role for *T. whipplei* and should therefore be removed to allow for efficient bacterial lysis and subsequent detection by qPCR. We assumed that processing would be sufficient to promote lysis of the bacteria during the denaturation step of qPCR. We used a pool of *T. whipplei*, *E. coli*, and an aliquot of purified human DNA to serve as reference calibration in real-time quantitative PCR (qPCR); One-third was treated with a glycosidase cocktail, the second-third with lysozyme, the last received no treatment. As shown in Fig. 4, the detection of *T. whipplei* through qPCR of the *whi2* gene improved upon pretreatment with a glycosidase cocktail relative to untreated controls (mean of Ct: 13.23 ± 0.93 vs 20.46 ± 0.13). However, treatment with lysozyme did not significantly alter the detection of *T. whipplei* (18.75 ± 0.50 vs 20.46 ± 0.13). Detection of *E. coli* using qPCR amplification of *OmpG* was more effective following lysozyme treatment (Ct: 12.80 ± 0.26 vs 19.60 ± 0.23), and moderately changed after treatment with glycosidase (Ct: 17.66 ± 0.26 vs 19.6 ± 0.23). The variation in the relative number of *T. whipplei* target sequences (=2^−ΔΔCT^) after glycan removal demonstrated enhanced sensitivity of *whi2* qPCR by 274-fold. Amplification of the control target, human β-actin, was nearly identical between glycosidase-treated samples (mean: 27.73 ± 0.37), lysozyme-treated samples (28.60 ± 0.26) and untreated samples (26.99 ± 0.03), indicating good inter-assay reproducibility. For each input, we observed no significant differences between each of the three individual qPCR reactions (less than 0.5 cycles, P < 0.01), a result that validated our assay.

**Figure 6:**
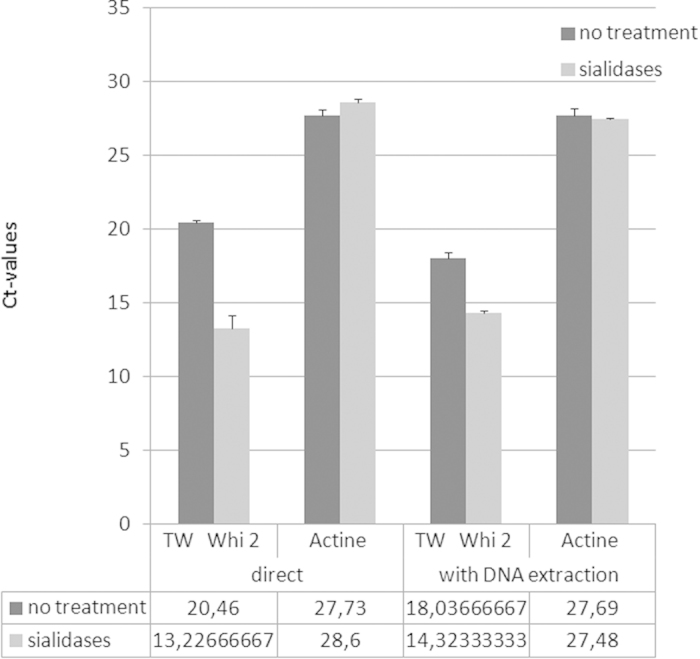
Effects of enzymatic treatment on RT-qPCR quantification of DNA. The graph represents the Ct-values obtained for RT-qPCR amplification of *T. whipplei whi2*, and the mammalian β-actin gene on untreated and treated samples as indicated in the right panel. Treatments were performed either directly analysed by RT-qPCR or after a DNA extraction as noted in the X-axis. (Significant differences between Ct-values of treated and untreated samples were indicated *P < 0.01).

Secondly, we wondered whether glycosidase treatment of the pooled *T. whipplei* and *E.coli* would facilitate extraction of bacterial DNA. The same enzymatic treatments as those described above were performed prior to DNA extraction of the pooled samples. Species-specific qPCR amplification was then performed in triplicate to monitor and quantify the detection of *T. whipplei*, *E.coli* and human DNA. Compared to untreated samples, we found that *T. whipplei* detection was more efficient after glycosidase treatment (14.32 ± 0.11 vs 18.03 ± 0.37) but unchanged after lysozyme treatment (18.87 ± 0.23 vs 18.03 ± 0.37). *E. coli* detection improved following lysozyme treatment (11.64 ± 0.38 vs 15.89 ± 0.12), but treatment with the glycosidase cocktail had a minimal effect (14.86 ± 0.29 vs 15.89 ± 0.12). The level of detection of the β-actin calibration control was equivalent for each test (no treatment: 27.69 ± 0.50; glycosidases: 27.48 ± 0.06; lysozyme: 28.02 ± 0.17), validating our assays (P < 0.01). As previously shown with direct qPCR, the removal of glycans facilitated the lysis of *T. whipplei* and extraction of its DNA, consequently increasing its detection by more than one log (Fig. 6). Overall, this demonstrated that *T. whipplei* is normally embedded in a complex glycan structure to protect it from lysis.

### Glycosidase treatment of clinical samples unmask cryptic *T. whipplei* DNA

**Figure 7:**
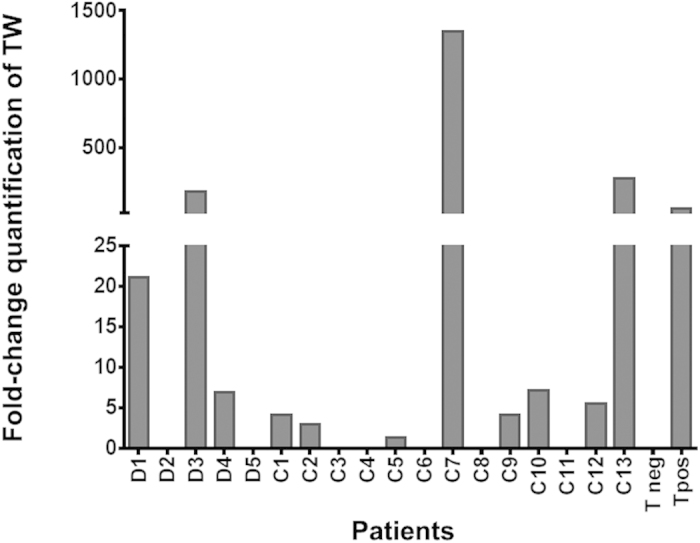
Glycosidase treatment of clinical samples unmasked the embedded *T. whipplei* and improved bacterial detection using qPCR assay. Extraction of total DNA from 5 DB (**a**) and 13 BAL (**b**) samples was carried out after glycosidase hydrolysis in parallel of the corresponding untreated samples. Each bar represents the fold-change in the relative abundances of whi2 sequences of *T. whipplei* of each sample normalized to the reference β-actin gene using the 2^−ΔΔCt^ method. All clinical information about patients (DB1 to DB5 and C1 to C13) are listed in Supplementary information S8. Samples from asymptomatic carrier patient C13 as well as a sample from a duodenal biopsy (DB Tpos) from patient presenting a persistent intestine WD were used as positive controls. A DB Sample from a patient without histological involvement and previously controlled in a qPCR assay showing no amplification of *T. whipplei* over 40 cycles was used as no template control (Tneg).

We investigated the effect of N- and O-glycan removal on the detection of *T. whipplei* from DB from Whipple’s patients already tested in our laboratory by conventional methods, with 5 presenting a positive PAS staining and a qPCR above the detection threshold (>34Ct) and 1 presenting both PAS staining and PCR positive (Tpos < 34Ct) and 1 from an uninfected individual (control Tneg). DNA extraction was performed on all untreated and glycosidase-treated samples, and triplicate *T. whipplei* qPCR reactions were carried out in parallel with qPCR of the β-actin gene, which served as an internal control for cell load. The concentration of the target *whi2* gene in each sample relative to that of the reference actin gene (=2^−ΔΔCt^) corresponds to the relative fold change quantification of bacteria for each condition. The removal of glycans increased the detection of *T. whipplei* sequences for the sample presenting a PAS positive and qPCR positive result (Tpos). Of the 5 samples presenting a previously positive PAS staining and a qPCR > 34Ct, the detection threshold of bacteria was improved for 3 samples and below of 34 Ct (DB1 = 21x; DB3 = 181x; DB4 = 7x) and 2 samples were left above the detection threshold of 34 Ct (DB2; DB5).The low variation in detection of the β-actin gene in triplicate samples validated our experimental data, which strongly indicate that treatment with glycosidases before DNA extraction reduces the threshold of detection in 4 samples (Fig. 7). Taken together, these results suggest that the previous discrepancies for the detection of *T. whipplei* between a positive immunohistochemistry staining and a qPCR > 34 Ct are outcome of inhibited proper extraction and/or detection of nucleic acid, due to biofilm in macrophages and not a complete eradication of the bacteria in the patient.

As previously, we investigated bacterial load based on DNA extraction performed on untreated and glycosidase-treated bronchoalveolar lavage (BAL) samples from 13 patients presenting with respiratory infection. We quantified the bacterial load by qPCR as described above for the DB. Figure 7 displays the effect of glycan hydrolysis on the detection. Despite the low level of human DNA in all the samples, bacterial detection was substantially improved in 2 out of 13 clinical samples (C7 = 1350x and C13 = 275x), while in 6 others, detection improved by between 7.5x and 1.5x. For the remaining 5 samples, bacterial detection was essentially unchanged. The bacterial detection from a previously diagnosed asymptomatic carrier increased by 275x. Interestingly, our method allowed us to detect *T. whipplei* DNA from one sample from patient presenting with pneumonia. We found minimal variation in detection of the β-actin gene across each set of triplicate reactions, validating our experimental data that strongly indicated treatment with glycosidases before DNA extraction reduced the threshold of detection in 7 out of 13 samples (Fig. 7).

## Discussion

We found that the biofilm surrounding *T. whipplei* within macrophage vacuoles is composed of complex polysaccharides that prevent detection of the bacterial DNA in many clinical samples. The hydrolysis of the biofilm by glycosidases improved access to specific *T. whipplei* probes and allowed for detection of bacteria. The large regions in samples with PAS-positive staining but no signal from DNA/RNA FISH probes represented secreted glycosylated *T. whipplei* antigens or dead bacteria, rather than live *T. whipplei*. This result established the presence of remnant bacilli in intercellular spaces, a result consistent with electron microscopy on ultrathin sections of intestinal biopsies from WD patients, which suggested autolytic activity of *T. whipplei*^30^. Our results also support previous work demonstrating a difference in Gimenez staining between the *T. whipplei* localized within macrophages and extracellular space, which was attributed to the enzymatic degradation of bacterial matrix^21^. On the other hand, our results revealing masked *T. whipplei* embedded inside infected cells from patients starting therapy and patients undergoing long-term therapy, suggest that the glycan biofilm confers a high level of resistance to antibiotic treatment, and supports the establishment of chronic and persistent infections. Reported cases of WD relapses occurring after long-term antibiotherapy support this hypothesis, albeit we cannot exclude the possibility of a re-infection as chronic asymptomatic carriers of *T. whipplei* can be colonized by different *T. whipplei* strains over time^31^. The resistance to antibiotic therapy has been intimately associated with biofilm formation^32^,^33^ and plays important roles in the chronic nature of the subsequent infections. Our model is that the excretion of *T. whipplei* which was previously identified in worsening WD^29^, is interrupted during the initiation of antibiotic treatment. Subsequently, only persistent bacteria reemerge from the intramacrophage biofilm compartment.

Here, we showed that the *T. whipplei* biofilm inside macrophage phagosomes confers protection and stability to mechanical, detergent, enzymatic and temperature stresses. These properties of surface-associated biofilms have previously been reported for numerous other bacteria, fungi, algae and archaea^22^,^26^,^32^. We propose that in Whipple’s disease, because of the intracellular replication niche, the concept of “compartment-associated” more appropriately fits this biofilm than does “surface-associated.”

Here, we were able to observe the presence of *T. whipplei* in the epithelial CK20^+^ cells. The bacterial RNA were found either with a diffuse staining in cytosol or with a granular pattern in CK20^+^ cells. The evidences of the presence of bacteria in few epithelial cells suggested a transcytosis of *T. whipplei* across epithelial layer. Then the clustered CD68^+^ macrophages around the infected epithelial cells are poised for the phagocytosis of *T. whipplei.* As *T. whipplei* survives by altering the phagosomal environment^34^, the macrophages surveillance led thereby to spread the infection. Aggregations of subepithelial macrophages containing bacterial RNA and DNA in numerous phagosomes support this hypothesis.

The deglycosylation process of the duodenal biopsies enhanced the colocalization of FISH and IF signals. Nevertheless, as we observed the IF staining fades after deglycosylation of biopsies. The difference probably resulted from a weaker IF staining of unglycosylated and deglycosylated proteins associated with a more diffuse DAPI staining. The efficacy of DAPI staining was partially reduced because of the denaturation step preceding the enzymatic deglycosylation reactions, generating single-stranded DNA which forms much weaker fluorescent complexes than with double-strand DNA. Altogether, sustained FISH signals emerged significantly by comparing the signal following glycosidase treatment to the signal of untreated samples. We propose that FISH should be carried out in parallel with the histological examination as an alternative approaches required for the evaluation of Whipple’s disease diagnostic and useful to follow up patient care.

Our study highlighted the presence of *T. whipplei* using qPCR in samples of patients without other evidence of Whipple’s disease. The presence of low bacterial loads in numerous samples from healthy patients was already mentioned^35^,^36^. The deglycosylation process before qPCR detection should be useful to study the prevalence in healthy population.

The development of original strategies to prevent and/or treat WD will likely advance significantly following elucidation of the nature of the biofilm in the pathogenesis of this systemic disease. The susceptibility of *T. whipplei* biofilms to glycosidases may have wider implications, as it is possible that inhibitors of PCR in tissues from leprosy patients could be similar to those described here^27^,^28^. Biofilms contains problematic physical inhibitors of the nucleic acid detection. We suggest that for live, biofilm-embedded bacteria, a simple step of glycan hydrolysis can be performed prior to nucleic acid extraction without significantly increasing the complexity of the extraction process.

## Materials and Methods

### Patients

This study was approved by the local ethics committee of IFR 48 (Marseille, France; n°07–035). The methods were carried out in accordance with the approved guidelines. Written informed consent was obtained from each patient for the use of information and data in the present study.

### *Tropheryma Whipplei* strains and culture

The *T. whipplei* Twist strain cultured both in MRC5 fibroblast cells^37^ and in axenic medium^8^ were tested in this study.

### Deglycosylation

Enzyme treatment to remove N- and O-linked carbohydrates from glycoproteins was performed as previously described^23^ with modifications (see supplement information).

### Fluorescent *In situ* hybridization assays and Confocal Microscopy

were performed as previously described^38^ with the modifications in supplement information.

### Co-localization analysis

We selected FISH images and their corresponding IF images for 18 biopsies. The co-localization of FISH and IF signals was identified and quantified using the free JACoP software^39^ and detailed in supplement information.

### Statistical analysis

The significance level throughout the study was set at *P* value < 0.01 using standard statistical software for analyses.

## Additional Information

**How to cite this article**: Audoly, G. *et al.* Deglycosylation of *Tropheryma whipplei* biofilm and discrepancies between diagnostic results during Whipple’s disease progression. *Sci. Rep.* **6**, 23883; doi: 10.1038/srep23883 (2016).

## Supplementary Material

Supplementary Information

Supplementary Video
