# Supplementary material for: Deglycosylation of Tropheryma whipplei biofilm and discrepancies between diagnostic results during Whipple’s disease progression
Source: Sci Rep. 2016 Mar 30;6:23883. doi: 10.1038/srep23883 (PMC4812295; doi:10.1038/srep23883)
Supplement: Supplementary Information [file srep23883-s1.pdf]

Deglycosylation of Tropheryma whipplei biofilm and discrepancies between diagnostic results during Whipple's disease progression

Gilles Audoly, Florence Fenollar, Jean-Christophe Lagier, Hubert Lepidi, Didier Raoult

Supplementary information:

Materials and Methods

Probe design: The 16S rRNA *T. whipplei* strain Twist5 (Accession number: NC_004572. GI: 32447382) probes were designed by using primer3-blast software and were then checked against the ribosomal database project (RDP; Michigan State University) to guarantee the specificity and sensitivity of the probes for *T. whipplei* rRNA (1). Two regions of *T. whipplei* were selected as target for hybridization of probes. The 16S rRNA probes sequences, labeling and their respective target are presented in Table 1 **(S7)**. The probes Tw16S-652, specific for *T. whipplei*, Eub338 and nonEub338 were previously designed and also used in our experiments (2). The amplification reactions of *T. whipplei* DNA probes were performed using Phusion High fidelity DNA polymerase following manufacturer recommendations (Finnzymes, ThermoFisher Scientific, Espoo Finland) with primers described in Table 1 (S7) and 25ng of genomic DNA of *T. whipplei twist*. We generated two DNA fragments of 6.7 and 6.4 kb corresponding to respectively TWT104 and TWT764 genes, from the wisp gene family. Each DNA fragment was then labeling by Nick translation with ARES DNA labeling

kit and coupled to Alexa 546 fluorophore (Invitrogen). Except non Eub, all the probes gave similar signals in replicative *T. whipplei*-infected MRC5 cells and in infected macrophages of duodenal biopsies from Whipple patients and purified *T. whipplei*. The wisp DNA probes were used to detect live and dead *T. whipplei*. The specificity of all probes was tested against irrelevant bacteria, briefly preparation of purified *Coxiella burnetii, E. coli, Bartonella Quintana, Rickettsia conorii* were spotted, onto glass slide, fixed before processed in *in situ* hybridization experiments.

Control experiments included RNase treatment of tissue or infected cells, to determine whether digesting the rRNA target prevented probe binding, and DNase treated samples were used to determine whether it prevented the Wisp genes probes binding.

Preparation of tissue sections and cells: Tissues studied included 35 duodenal biopsy specimens from patients with classic Whipple's disease, characterized by positive periodic acid-Schiff, immunohistochemistry, specific positive *T. whipplei* polymerase chain reaction (PCR), and 4 biopsy specimens from patients not related to a Whipple's disease, all of them have been described elsewhere(3) **.** Five to 20 tissue sections for one patient were included in our hybridization *in situ* experiments (Supplementary Table S8).

Deglycosylation: Samples were denatured using Glycoprotein Denaturing Buffer (New England Biolabs) at 94°C for 5 min. Samples were then incubated for 10 h at 37°C with 5 units of each glycosidase per biopsy sample. The enzymatic cocktail consisted of the recombinant endoglycosidase H (PNGase H) from *Streptomyces plicatus* (Sigma-Aldrich), the recombinant EndoHf (New England Biolabs), and the endo-O-Glycosidase from *streptococcus pneumoniae* (Sigma-Aldrich). Negative controls were denatured under the same conditions and were then incubated in the Glycoprotein Denaturing Buffer alone, without any

glycosidase. Deglycosylation treatments were checked by parallel experiments with *T. whipplei* preparations, and modifications of electrophoretic mobility of all proteins were analyzed by SDS-page and western blotting with specific hyper-immune mouse serum directed against *T. whipplei* (4).

Fluorescent In situ hybridization: were performed as previously described (5) (6) with following modifications. Glass slides with *T. whipplei* infected MRC5 cells, uninfected cells or duodenal biopsies from Whipple patients were included in our experiments. Both the prehybridization and the hybridization were performed in Hybridizer (Dako, Trappes France) in HB buffer: 4X SSC (1 SSC is 15 mM sodium citrate plus 0.15 M sodium chloride), 10% dextran sulfate, 1X Denhardt'solution, 0.5mg/ml *E.coli* tRNA, and 25% formamide with diluted probe to100 nM. Slides were prehybridized at 65°C for 15 min followed by hydridization at 37°C overnight. Then, we performed three stringent washes in 4XSSC, 2XSSC-0.1% Triton X-100 and 0,5XSSCT slides at room temperature.

Immunofluorescence Assays: After the stringent washes, slides were immerged in blocking reagent (PBS- 3% BSA- 0.5% Triton X-100) for 30 min at RT and then incubated for 1 h at 37°C with a hyperimmunized mouse serum to *T. whipplei* that is currently used in our lab for diagnostic assays (29) or monoclonal anti-CD68 (Dako) to detect macrophages in duodenal biopsies, or monoclonal anti-CK20 (Dako) to detect epithelial cells. Unbound antibody was removed by three washes in PBS- 0.5% Triton X-100 for 10 min each. Then, the samples were incubated with appropriate conjugated goat anti-mouse coupled to an Alexa 647 or Alexa 555 (1:1000; Life Technologies, Saint-Aubin France). After 3 further washings, the slides were air-dried and mounted with 4′,6-diamidino-2-phenylindole (DAPI) from a ready-to-use solution (ProLong Gold Antifade Reagent; Molecular Probes). Following *in situ* hybridization, cells were visualized under a Leica DM6000 Upright fluorescence microscope

with appropriate filter sets for fluorescence, and with X10, X20, X40 plan Apochromat objective and an X100 oil immersion objective. The imaging system is driven by MetaMorph® (Molecular Devices, Sunnyvale, CA, USA) for multi-dimensional image acquisition. Confocal analysis of samples was done using a Leica TCS 4DA confocal microscope. The laser emission lines used were 488 nm and 568 nm. Image analysis were done using Leica application suite AF version 2.4.1 (Leica Microsystems CMS Gmbh, Germany). More than 250 hybridizations were done using MCR5 infected cells, uninfected cells, sections of tissues from patients with Whipple's disease and normal biopsy specimens, and each experiments was viewed at several magnifications, ranging from objectives X10 to X100.

Co-localization analysis: The co-localization of FISH and IF signals was identified and quantified using the free JACoP software (7). This protocol helped ensure reliability of colocalization coefficient calculations and avoid bias of visual interpretation. Pearson's correlation coefficient is a quantitative measurement that estimates the degree of overlap between fluorescence signals obtained in two channels. The Pearson's correlation values range from −1.0 to 1.0, where 1 indicates perfect co-localization, 0 indicates no significant correlation and −1.0 indicates complete separation of the two signals. The Coste's program automatically generated the threshold for each channel with 200 randomization rounds and chose a *P*-value of >95% to obtain a significant statistical distribution to more reliable elimination of false positives. The numerical values of co-localization coefficients were interpreted using a unified approach (8) and were classified as very strong (1.0 to 0.85), strong (0.85 to 0.49), moderate (0.49 to 0.1), weak (0.09 to -0.26) and very weak (−0.27 to −1).

DNA extraction: A 200 µl aliquot of standardized suspensions from bronchoalveolar lavages (BAL) and approximately 100 µg of DB were nitrogen frozen and quickly mechanically disrupted with an equal volume of glass beads (<106 µm) prior to direct digestion of glycans as described in the “deglycosylation” section. DNA extraction from biopsy samples was carried out with the EZ1Qiagen kit following the manufacturer’s recommendations.

RT-qPCR: Total bacterial load was analyzed by real-time quantitative PCR using a specific primer-probe set targeting the *T. whipplei whi 2* genes, the human actin gene, using the CFX96™ Real-Time PCR System (Bio-Rad). The analytical sensitivities of the real-time PCR targeting these genes were determined in triplicate reactions using ten-fold serial dilutions of a known titration of *T. whipplei* from axenic culture (9;10). All samples were processed in triplicate.

.

Reference List

(1) Cole JR, Wang Q, Cardenas E, et al. The Ribosomal Database Project: improved alignments and new tools for rRNA analysis. Nucleic Acids Res 2009 Jan;37(Database issue):D141-D145.

(2) Fredricks DN, Relman DA. Localization of Tropheryma whippelii rRNA in tissues from patients with Whipple's disease. J Infect Dis 2001 Apr 15;183(8):1229-37.

(3) Lepidi H, Fenollar F, Gerolami R, et al. Whipple's disease: immunospecific and quantitative immunohistochemical study of intestinal biopsy specimens. Hum Pathol 2003 Jun;34(6):589-96.

(4) Fenollar F, Amphoux B, Raoult D. A paradoxical Tropheryma whipplei western blot differentiates patients with whipple disease from asymptomatic carriers. Clin Infect Dis 2009 Sep 1;49(5):717-23.

(5) Audoly G, Vincentelli R, Edouard S, et al. Effect of rickettsial toxin VapC on its eukaryotic host. PLoS One 2011;6(10):e26528.

(6) Atieh T, Audoly G, Hraiech S, et al. Evaluation of the diagnostic value of fluorescent in situ hybridization in a rat model of bacterial pneumonia. Diagn Microbiol Infect Dis 2013 Aug;76(4):425-31.

(7) Bolte S, Cordelieres FP. A guided tour into subcellular colocalization analysis in light microscopy. J Microsc 2006 Dec;224(Pt 3):213-32.

(8) Zinchuk V, Wu Y, Grossenbacher-Zinchuk O. Bridging the gap between qualitative and quantitative colocalization results in fluorescence microscopy studies. Sci Rep 2013;3:1365.

(9) Fenollar F, Fournier PE, Raoult D, Gerolami R, Lepidi H, Poyart C. Quantitative detection of Tropheryma whipplei DNA by real-time PCR. J Clin Microbiol 2002 Mar;40(3):1119-20.

(10) Fenollar F, Fournier PE, Robert C, Raoult D. Use of genome selected repeated sequences increases the sensitivity of PCR detection of Tropheryma whipplei. J Clin Microbiol 2004 Jan;42(1):401-3.

Supplementary Table 7

Primer/probe name	Target	5'modification	sequence	reference
TWT-S1	Forward Wisp104	n.a.	GTGTCCTGCGTATTG TGTGC	this study
TWT-R5110	Reverse Wisp104	n.a.	GGCTGACCTGTGTT CCTAGC	this study
TWT-S104	Forward Wisp764	n.a.	CAGGGTTGTGGCTG TACCTT	this study
TWT-R104	Reverse Wisp764	n.a.	GTAGACCTACCGGG ACGTGA	this study
TW 16S-1402	16S rRNA	Alexa 488	CGGGTGTTACCAGC TTTCAT	this study
TW 16S-617	16S rRNA	Alexa 488	CCCTATCGCACTCTA GCCTG	this study
TW 16S-652	16S rRNA	Alexa 488	TTCCGCTCTCCCCTA TCGCACTCT	Fredricks and Relman *J Infect Dis.* *(2001) 183 (8): 1229-1237*
Eub 338	16S rRNA	Alexa 594	GCTGCCTCCCGTAG GAGT	Amann RI, *Microbiol* 1990; 56: 1919–1925.
Non eub	antisens 16S rRNA	Alexa 594	ACTCCTACGGGAGG CAGC	Amann RI, *Microbiol* 1990; 56: 1919–1925.

Supplementary Table 8

list of patient's information included in the present study.

Code	**Type of sample**	**Age (y)**	**Gender**	**Diagnosis**	**Treatment status**	**Duration of treatment (w/m/y)**	**supplementary information**
A1	Small bowel biopsy	76	M	CWD	Doxycycline + hydroxychloroquine	14 m	Persistent intestin[illegible] and jejunum WD, PCR>34Ct
A2	Small bowel biopsy	54	M	CWD	Doxycycline + hydroxychloroquine	26 m	Negative IHC on duodenal biopsy, PCR>34Ct
A3	Small bowel biopsy	44	F	CWD	Doxycycline + hydroxychloroquine	5 y	Persistent intestine WD, duodenal biopsy with a negative IHC and a PCR>34Ct
A4	Small bowel biopsy	67	M	CWD	Trimethoprim-sulfamethoxazole	9 y	Persistent intestine WD with an infiltration of submucosa, duodenal biopsy with a negative IHC and a PCR>34Ct
A5	Small bowel biopsy	54	M	CWD	Trimethoprim-sulfamethoxazole	5 m	Persistent intestine WD, PCR>34Ct
A6	Small bowel biopsy	54	M	CWD	Trimethoprim-sulfamethoxazole	5 m	Persistent intestine and jejunum WD PCR>34Ct
A7	Small bowel biopsy	76	M	CWD	Doxycycline + hydroxychloroquine	2 y	Persistent intestine and jejunum WD, PCR>34Ct
A8	Small bowel biopsy	80	M	CWD	Trimethoprim-sulfamethoxazole	2 y	Persistent duodenal and jejunum WD, PCR>34Ct
A9	Small bowel biopsy	51	M	CWD	doxycycline + hydroxychloroquine	4 y	Persistent duodenal WD, PCR>34Ct
A10	Small bowel biopsy	31	F	CWD	Doxycycline + hydroxychloroquine	2 y	Persistent duodenal and jejunum WD, PCR>34Ct
A11	Small bowel	1	M	CWD	nd	1 y	Persistent intestine WD,

	biopsy						PCR>34Ct
A12	Small bowel biopsy	74	M	CWD	Trimethoprim-sulfamethoxazole	2 y	Persistent intestine WD, PCR>34Ct
A13	Small bowel biopsy	62	F	CWD	Doxycycline + hydroxychloroquine	1 m	WD, PCR>34Ct
A14	Small bowel biopsy	78	M	CWD	Trimethoprim-sulfamethoxazole since 2001, then Doxycycline + hydroxychloroquine since 2006	11 y	Persistent of rare IHC+ macrophages
A15	Small bowel biopsy	nd	M	CWD	Trimethoprim-sulfamethoxazole	-	WD, positive PCR
A16	Small bowel biopsy	63	M	CWD	Doxycycline + hydroxychloroquine	-	Persistent intestine WD, positive PCR
A17	Small bowel biopsy	27	F	CWD	nd	2 y	Persistent intestine WD, PCR>34Ct
A18	Small bowel biopsy	68	M	CWD	Trimethoprim-sulfamethoxazole since 2001, then Doxycycline + hydroxychloroquine since 2006	11 y	Persistent intestine WD, PCR>34Ct
A19	Lymph node	67	F	inflammatory anemia lymphadenopathy , polyarthritis	NSAIDs, corticotherapy No antibiotic therapy	-	Diagnosis of WD, blood and lymph node with positive PCR
B1	Small bowel biopsy	43	M	No histological involvement	No antibiotic therapy	-	
B2	Small bowel biopsy	78	M	No histological involvement	No antibiotic therapy	-	
B3	Small bowel biopsy	84	M	No histological involvement	No antibiotic therapy	-	
C1	BAL	4	M	Chronic granulomatous disease	Trimethoprim-sulfamethoxazole + itraconazole + ceftriaxone	nd	
C2	BAL	51	M	AIDS patient	Trimethoprim-sulfamethoxazole + HAART	nd	
C3	BAL	4	M	Leukemia	Trimethoprim-sulfamethoxazole	1 m	
C4	BAL	11	F	Lung nodule	No antibiotic therapy	-	
C5	BAL	54	F	Pneumonia	Ceftriaxone	7 d	
C6	BAL	78	M	Pneumonia	Piperacilline-tazobactam + azithromycine	7 d	
C7	BAL	32	M	Pneumonia	Ceftriaxone + Levofloxacine	7 d	
C8	BAL	43	F	Pneumonia	Ceftriaxone + Levofloxacine	7 d	
C9	BAL	55	M	Lung nodule	Ceftriaxone	7 d	
C10	BAL	47	M	Pneumonia following	Ceftriaxone + Levofloxacine	14 d	

				pulmonary transplantation			
C11	BAL	49	F	Pneumonia	No antibiotic therapy	nd	PCR>34Ct
C12	BAL	51	M	Pneumonia	Ceftriaxone + Azithromycine	nd	
C13	SAL	52	M	Asymptomatic carriage	No antibiotic therapy	-	Positive PCR
DB1	Small bowel biopsy	62	M	CWD	Doxycycline + hydroxychloroquine	1 y	Persistent intestine WD, PCR>34Ct
DB2	Small bowel biopsy	57	M	CWD	Doxycycline + hydroxychloroquine	1y	Persistent intestine WD, PCR>34Ct
D3	Small bowel biopsy	55	M	CWD	Doxycycline + hydroxychloroquine 1 y, lifelong doxycycline	4 y	Persistent intestine WD, PCR>34Ct
DB4	Small bowel biopsy	56	M	CWD	Doxycycline + hydroxychloroquine	5 m	Persistent intestine WD, PCR>34Ct
DB5	Small bowel biopsy	56	M	CWD	Doxycycline + hydroxychloroquine	nd	Persistent intestine WD, PCR>34Ct
Tneg	Small bowel biopsy	65	M	No histological involvement	No antibiotic therapy	-	-
Tpos	Small bowel biopsy	53	M	CWD	Doxycycline + hydroxychloroquine	1 y	Persistent intestine WD, positive PCR

Table 2: list of patients included in the present study. A1 to A18= Small bowel biopsy from classical WD (CWD) patients (figure 1, 2, 5), A19 lymph node (figure 3), B1 to B3 = small bowel biopsy from patients W/O WD (figure 1, 2, 5). C1 to C13 = BAL or SAL and D1 to D7 =Small bowel biopsy (figure 7). M: male, F: female, BAL broncholaveolar lavage, SAL saliva, DUB= duodenal biopsy, IHC: Whipple immunohistochemistry, PCR was considered positive if both PCR assays were <34Ct for Whi2/Whi3 amplifications, Ct =cycle threshold, sample with a PCR>34Ct is considered negative, nd: not documented, -: not applicable, y= years, m= months, d=days

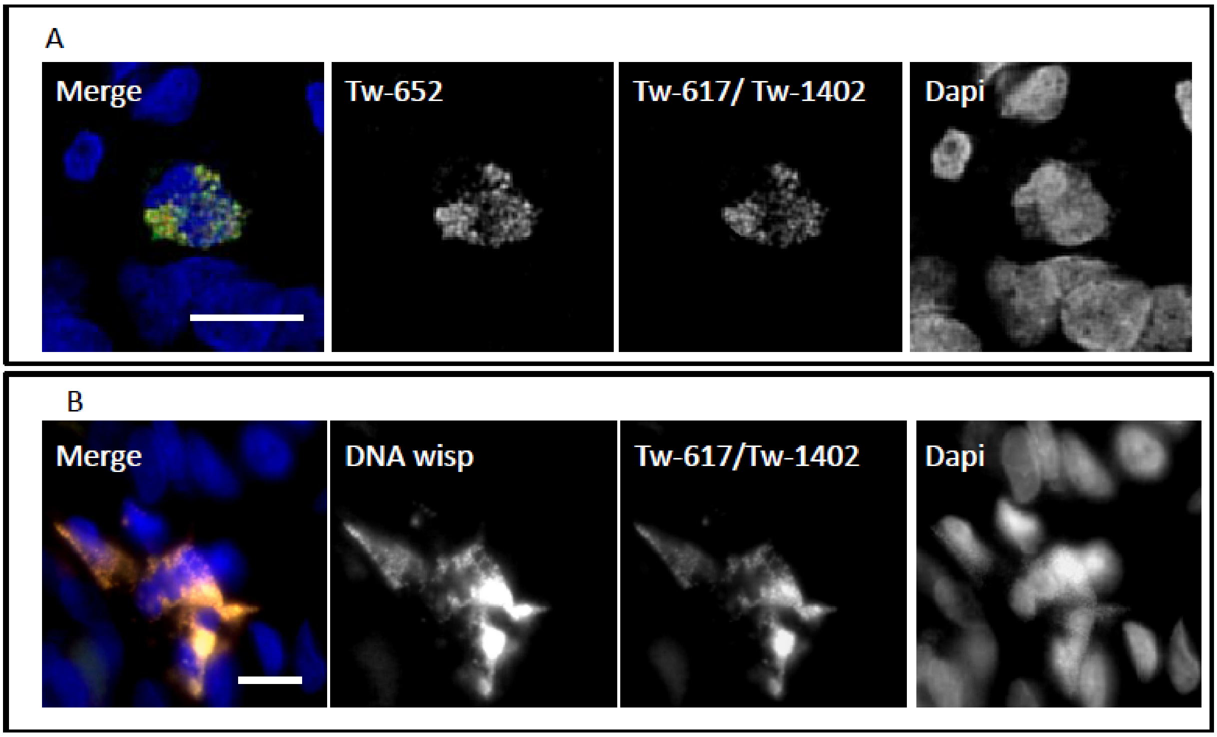

Supplementary figure 1: Confocal analysis of duodenal biopsy from a patient presenting Whipple disease. Probes used to detect *T. whipplei* are indicated in the upper left corner of each image; nuclei are counterstained with DAPI. The merge image and the individual emission channels for each fluorophore are presented. The merge image in A) shows a co-localization of the probes Tw-617/Tw-1402 in green with the Tw-652 in red around nucleus of one infected cells. FISH probes are concentrated in vacuoles which appear in yellow or white color surrounding nucleus, note that most of the bacterial DNA present in vacuoles are stained by DAPI suggesting a high concentration of bacteria vacuoles. The merge image in B) presents a co-localization of the probes Tw-617/Tw-1402 in green and wisp DNA probes in red around nucleus of infected cells, confirming the specificity of Tw-617 and Tw-1402 staining. Scale bars = 10 µm.

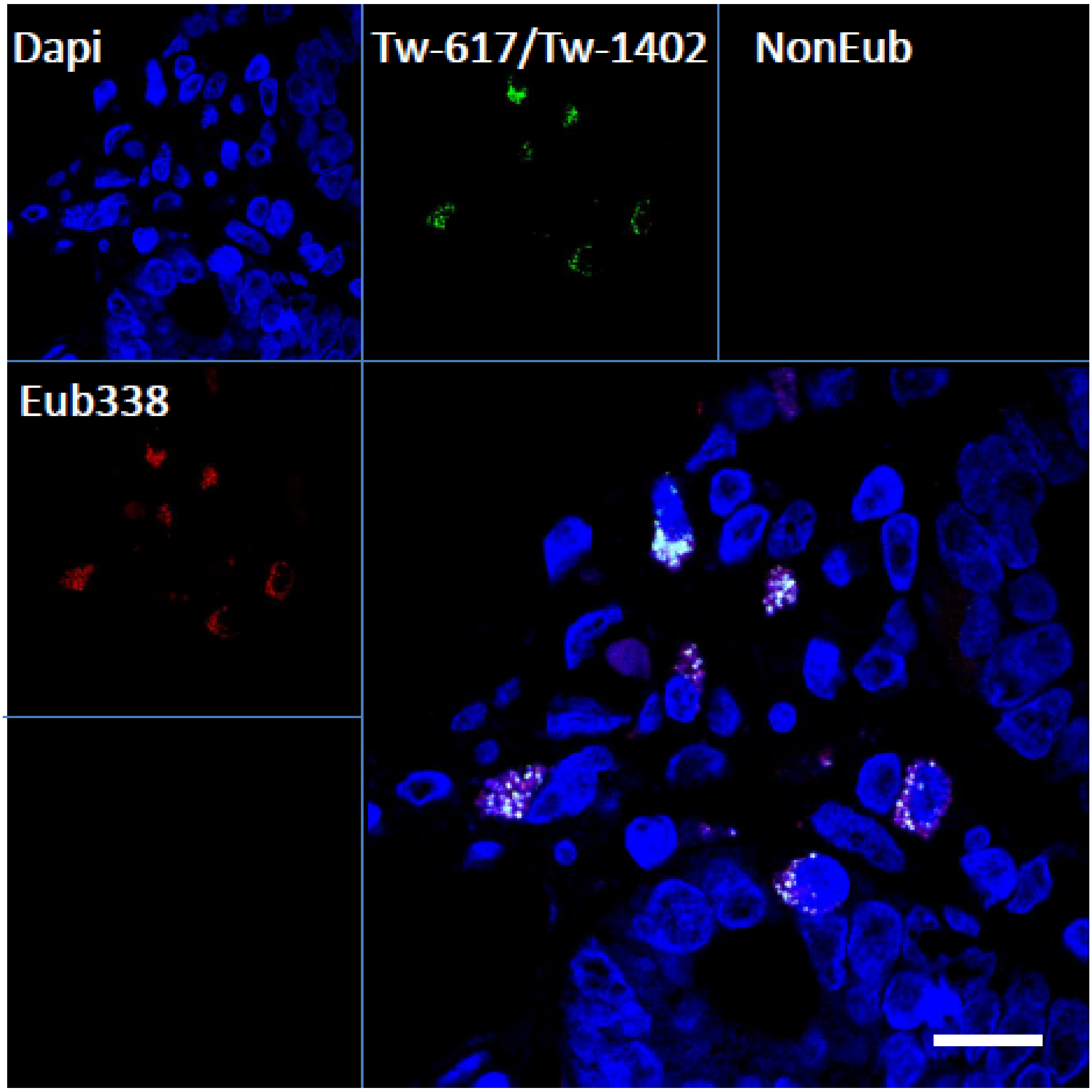

Supplementary figure 2: Confocal analysis of a duodenal biopsy from a patient presenting Whipple disease. Probes used to detect *T. whipplei* are indicated in the upper left corner of each image. Each field is shown with 16S rRNA FISH in green and NonEub channel corresponding to Alexa 546, Eub 338 to channel corresponding to far red Alexa-647. Nuclei are counterstained with DAPI. Scale bars = 20µm.

Supplementary video 3: The video of a compiled confocal Z-stack from a Whipple duodenal biopsy hybridized with green fluorescent probes directed against *T. whipplei* 16S rRNA

probes Tw-617/Tw-1402. The video reveals macrophages with infected vacuoles surrounding the DAPI-stained nucleus. Scale bar is in the bottom left of the video, and the Z-stack slices are in nm.

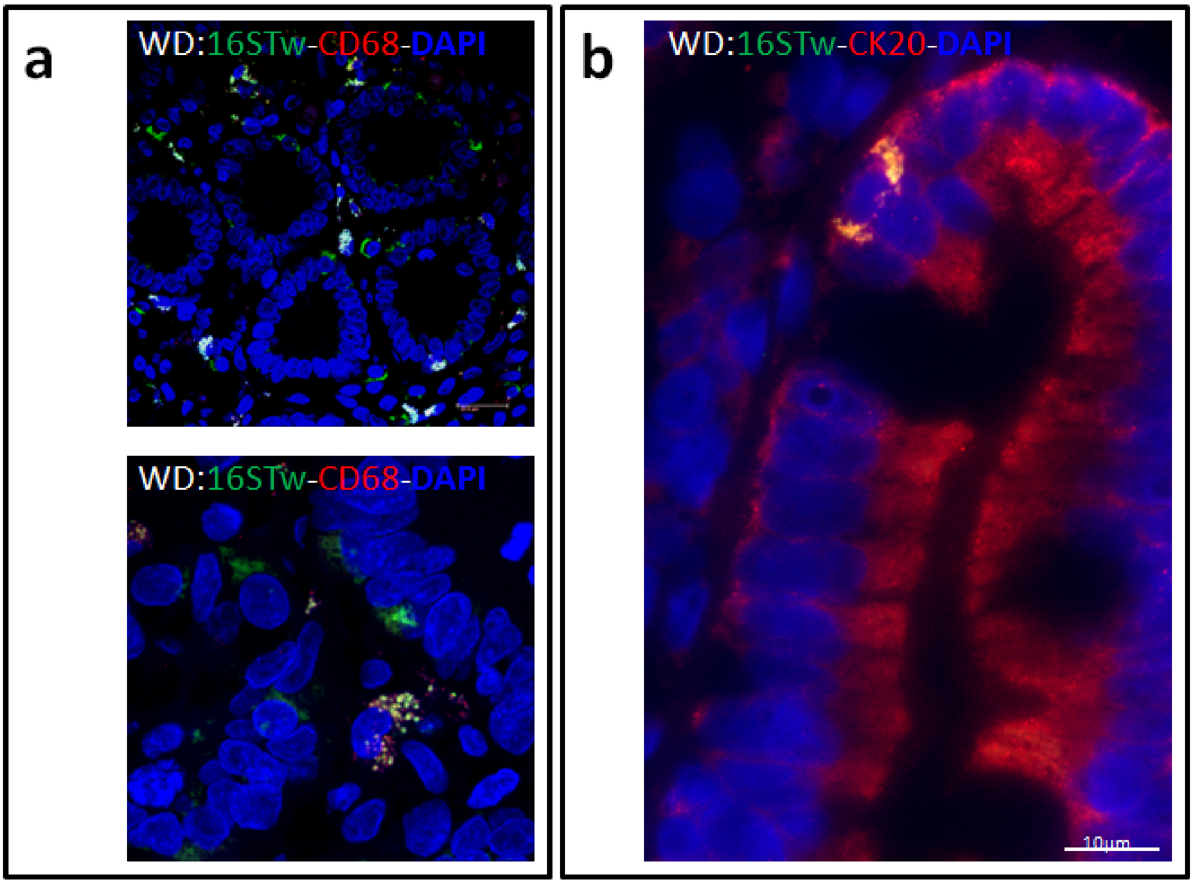

Supplementary figure 4: Confocal microscopy analysis of a duodenal biopsy from a patient presenting Whipple disease. (a) Probes used to detect *T. whipplei* are Tw-617/Tw- 1402 (green) associated to IF with anti-CD68. (b) Probes used to detect *T. whipplei* are Tw-617/Tw- 1402 (green) associated to IF with anti- CK20 in red. Nuclei are counterstained with DAPI. In A, Scale bars = 100µm, in B=30 µm, in C= 10µm.

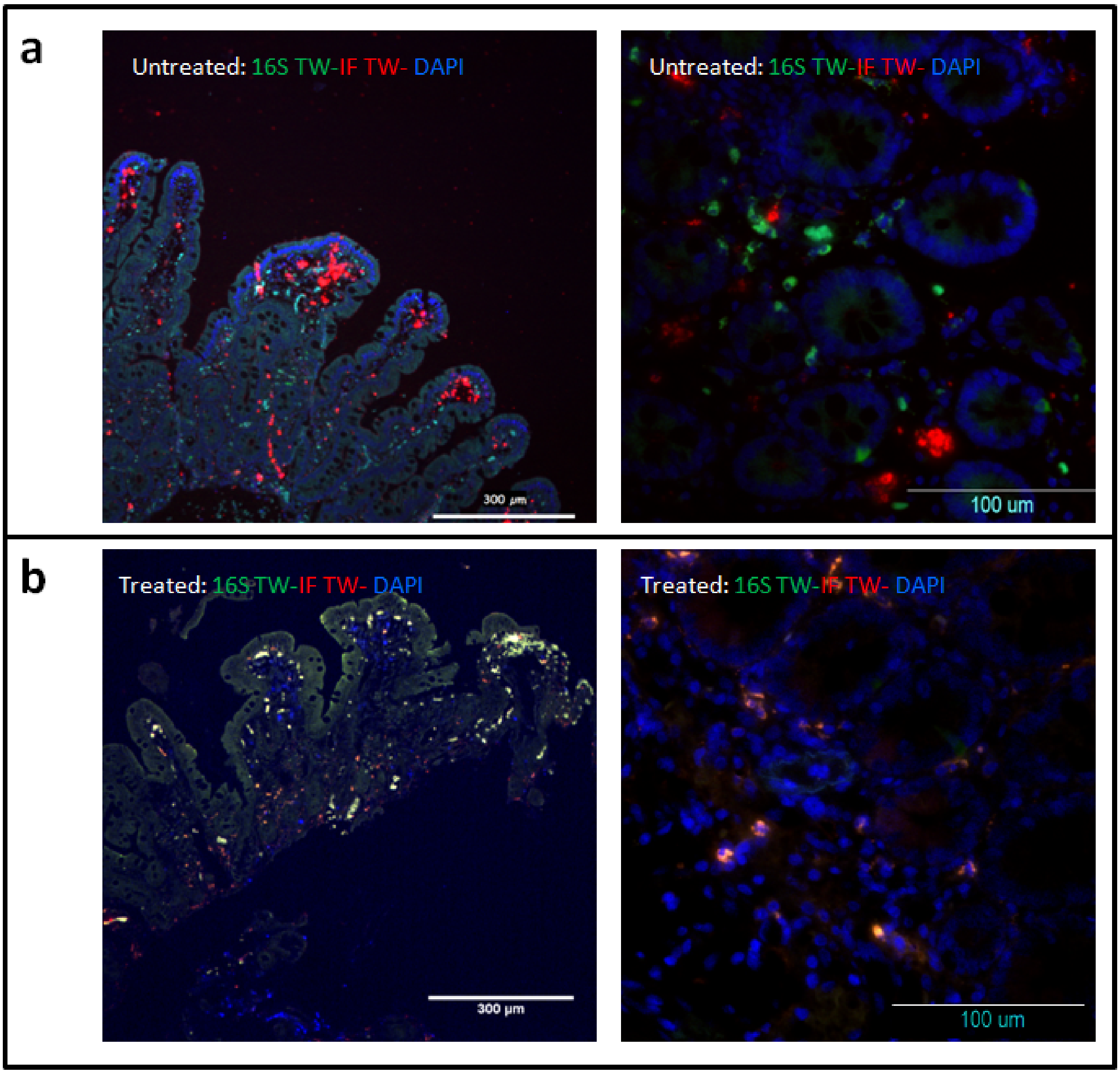

Supplementary figure 5: Effect of deglycosylation on *T. whipplei* detection by FISH/IF assays of a duodenal biopsy from patients presenting Whipple disease. We present the FISH and IF images from consecutive sections of the same duodenal biopsy. Probes used to detect *T. whipplei* are 16S rRNA Tw-617/Tw- 1402 (green), followed by an immunofluorescence staining by *T. whipplei*-specific antibodies in red. Nuclei are counterstained with DAPI. (**a**) without deglycosylation process (untreated);(**b**) slides after a deglycosylation process (treated). Scale bar is indicated on each image.

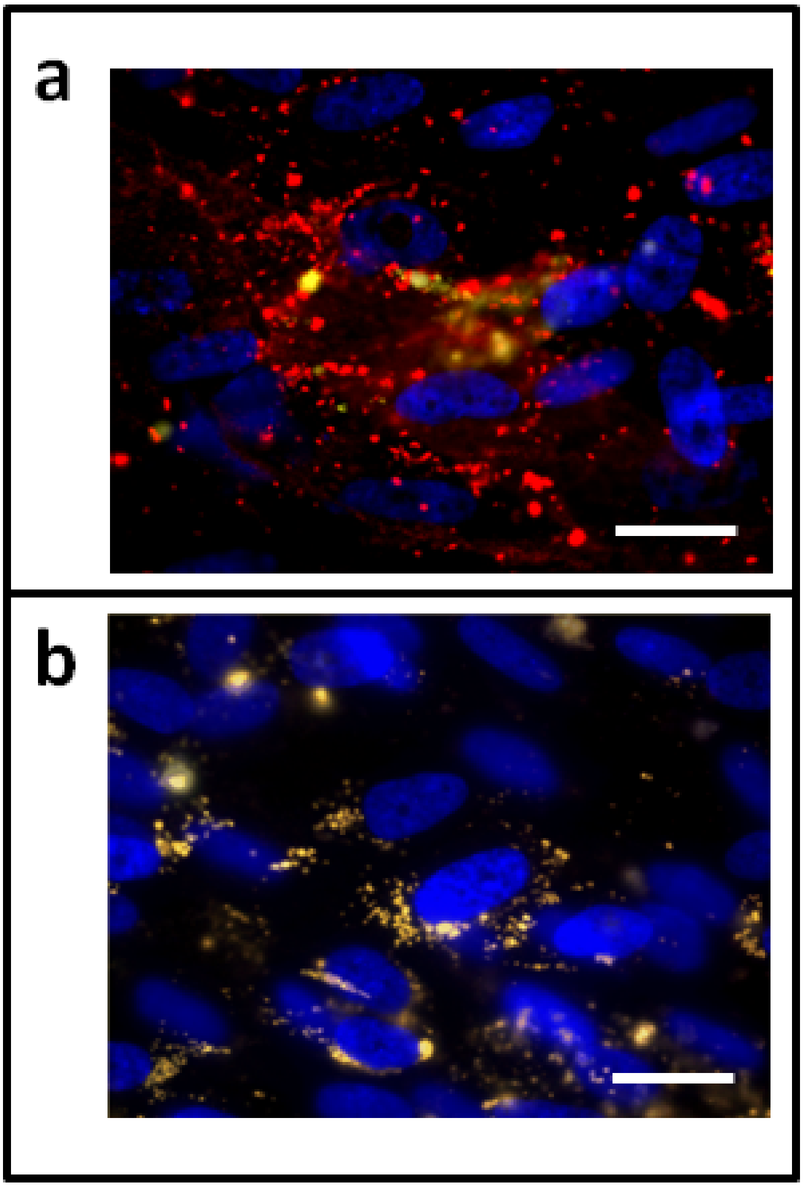

Supplementary figure 6: Glycosidases treatment of MRC5 infected cells increase T. whipplei detection and colocalization by FISH/IF assays. Probes used to detect *T. whipplei* are 16S rRNA Tw-617/Tw- 1402 (green), followed by an immunofluorescence staining by *T. whipplei*-specific antibodies in red. Nuclei are counterstained with DAPI. **(a**) without deglycosylation process (untreated); **(b**) after a deglycosylation process (treated). Scale bar=10µm.
